# Supplementary material for: VE-cadherin in arachnoid and pia mater cells serves as a suitable landmark for in vivo imaging of CNS immune surveillance and inflammation
Source: Nat Commun. 2023 Sep 20;14:5837. doi: 10.1038/s41467-023-41580-4 (PMC10511632; doi:10.1038/s41467-023-41580-4)
Supplement: Supplementary file 1 — Supplementary information [file 41467_2023_41580_MOESM1_ESM.pdf]

Supplementary Information

VE-cadherin in arachnoid and pia mater cells serves as a suitable landmark for *in vivo* imaging of CNS immune surveillance and inflammation

Josephine A. Mapunda[1, †] , Javier Pareja[1,†], Mykhailo Vladymyrov[1], Elisa Bouillet[1], Pauline Hélie[1], Petr Pleskač[1], Sara Barcos[1], Johanna Andrae[2], Dietmar Vestweber[3], Donald M. McDonald[4], Christer Betsholtz[2,5], Urban Deutsch[1], Steven T. Proulx[1], Britta Engelhardt[1]

[1]Theodor Kocher Institute, University of Bern, Bern, Switzerland, [2]Department of Immunology, Genetics and Pathology, Uppsala University, Uppsala, Sweden, [3]Max-Planck- Institute for Molecular Biomedicine, Münster, Germany, [4] Cardiovascular Research Institute, UCSF Helen Diller Family Comprehensive Cancer Center, and Department of Anatomy, University of California San Francisco, USA, [5]Department of Medicine-Huddinge, Karolinska Institute, Campus Flemingsberg, Huddinge, Sweden

[†]equal contribution

Running title: VE-cadherin landmarks leptomeninges

Keywords: arachnoid mater, pia mater, leptomeninges, VE-cadherin, two-photon imaging

Correspondence:

Prof. Dr. Britta Engelhardt
Theodor Kocher Institute
University of Bern
Freiestrasse 1
3012 Bern
Switzerland
E-Mail: britta.engelhardt@unibe.ch

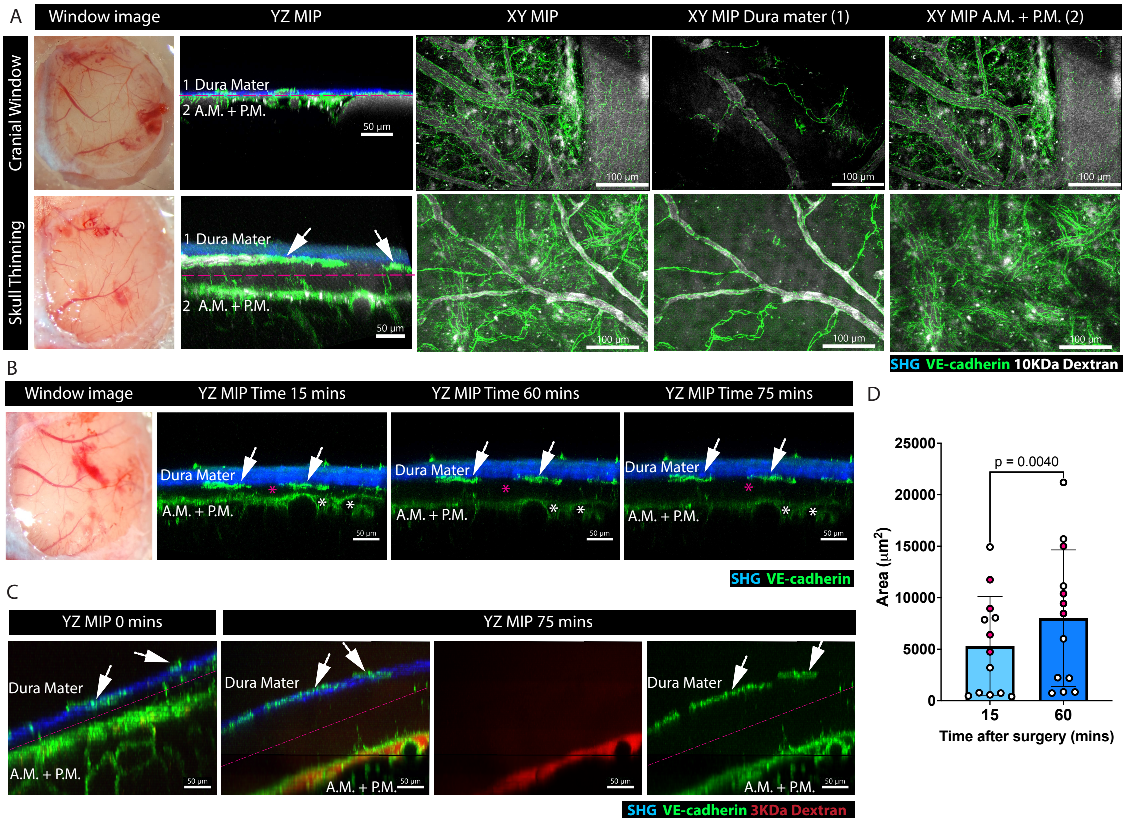

Supplementary Figure 1: The method of brain imaging impacts on the structure of the meningeal layers
A) Representative images from the surgical preparations and respective 2P-IVM imaging of the brains of VE-cadherin-GFP mice after acute cranial window or skull thinning preparation. Mice were injected intraarterially with a 10kDa-AF647 dextran tracer to label the lumen of barrier forming blood vessels and the dura mater lacking a BBB. The VE-cadherin-GFP signal from the blood vessels and arachnoid and pia mater is seen in green. SHG depicts the dura mater in the cranial window preparation and the remnants of the bone and dura mater in the skull thinning preparation. Cropped XY MIP images are shown on the right from the regions of the full Z-stack as well as the Z-stacks above (1) and below (2) the magenta dashed line in the YZ MIP images. White arrows point to dural vessels. Data are representative of three independent experiments per condition. **B)** Representative 2P-IVM images from the brain of a healthy VE-cadherin-GFP mouse taken 15, 60 and 75 mins after skull thinning. The VE-cadherin-GFP signal from the blood vessels and arachnoid and pia mater is seen in green. SHG depicts the remnants of the bone and the dura mater. White asterisks indicate the expected SAS, magenta asterisks indicate a subdural space that is widening over time. White arrows point to dural vessels. Data is representative of three independent experiments. **C)** Representative 2P-IVM images from the brain of a healthy VE-cadherin-GFP mouse after acute cranial window preparation. Prior to the cranial window preparation, a tracer-filled cannula was implanted into the cisterna magna. During 2P-IVM, the mouse was injected with 2.5µl of 3kDa TRITC dextran at a rate of 1µl/min using a syringe pump. The VE-cadherin-GFP signal from the blood vessels and arachnoid and pia mater is seen in green. SHG depicts the dura mater. Images were taken 0 and 75 minutes after tracer injection. After 75 minutes the cisterna magna injected tracer is visible in the SAS (red) and a widened subdural space is observed. White arrows point to dural vessels. **D)** Quantification of the subdural space area from brain 2P-IVM image-cross-sections after acute cranial window (white dots) and skull thinning preparations (magenta dots) in healthy VE-cadherin GFP knock-in mice. Data were pooled from 13 videos from 11 independent experiments (4 skull thinning preparations and 7 cranial

windows) and analysed using two-sided paired parametric T-test and shown as mean ± SD. Source data are provided as a Source Data file.

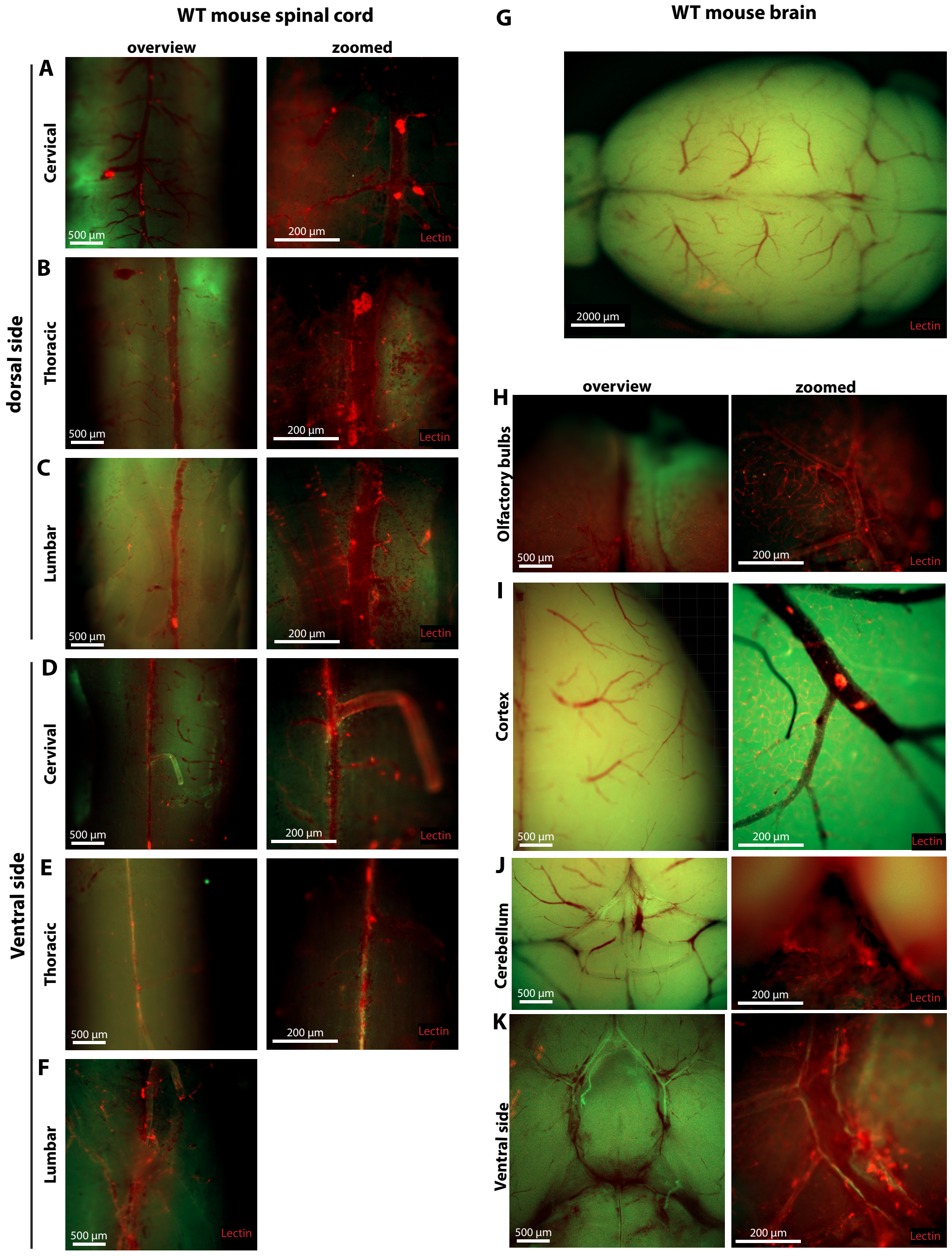

Supplementary Figure 2. Meningeal layers covering the brain and spinal cord of a wild-type C57BL/6J mouse Representative images of *ex-vivo* imaging of the leptomeningeal layers covering the different regions of the brain and spinal cord of a wild type C57BL/6J mouse taken by the Axiozoom fluorescence microscope. The green signal is due to tissue auto fluorescence. Lectin vascular tracer was injected intravenously to visualize the blood vessels (red). A-F) Overviews (left) and high magnification (right) representative images of the leptomeningeal layer covering the A) dorsal and D) ventral cervical, B) dorsal and E) ventral thoracic, and C) dorsal and F) ventral lumbar regions of the spinal cord. G) Representative image of an overview of the leptomeningeal layer covering the whole brain surface dorsally. Blood vessels are visible in red. H-K) Overviews (left) and high magnification (right) representative images of the leptomeningeal layer covering the dorsal side

of H) the olfactory bulbs I) the cortex J) the cerebellum, and K) the ventral side of the brain. Data is representative of three independent experiments.

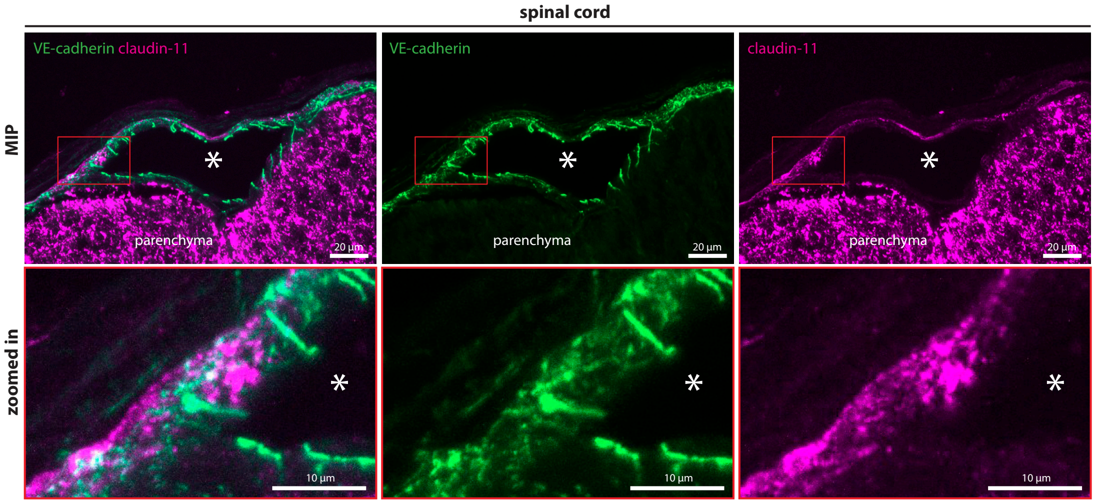

Supplementary Figure 3. Cells of the arachnoid and pia mater of the spinal cord express VE-cadherin
Immunofluorescence staining and confocal imaging of 20 µm thick sections of the decalcified vertebral column collected from healthy VE-cadherin-GFP knock-in mice immunostained for claudin-11 (magenta). Claudin-11 mostly appears as a thin upper layer of the arachnoid mater and does not colocalize with the VE-cadherin-GFP (green). No claudin-11 staining was detected at the level of the pia mater. Claudin-11 immunostaining in the spinal cord white matter detects claudin-11^{+} tight junctions of the myelin sheaths. Asterisks indicate the lumen of the dorsal spinal cord vein. Magnification 63x. Data is representative of three independent experiments.

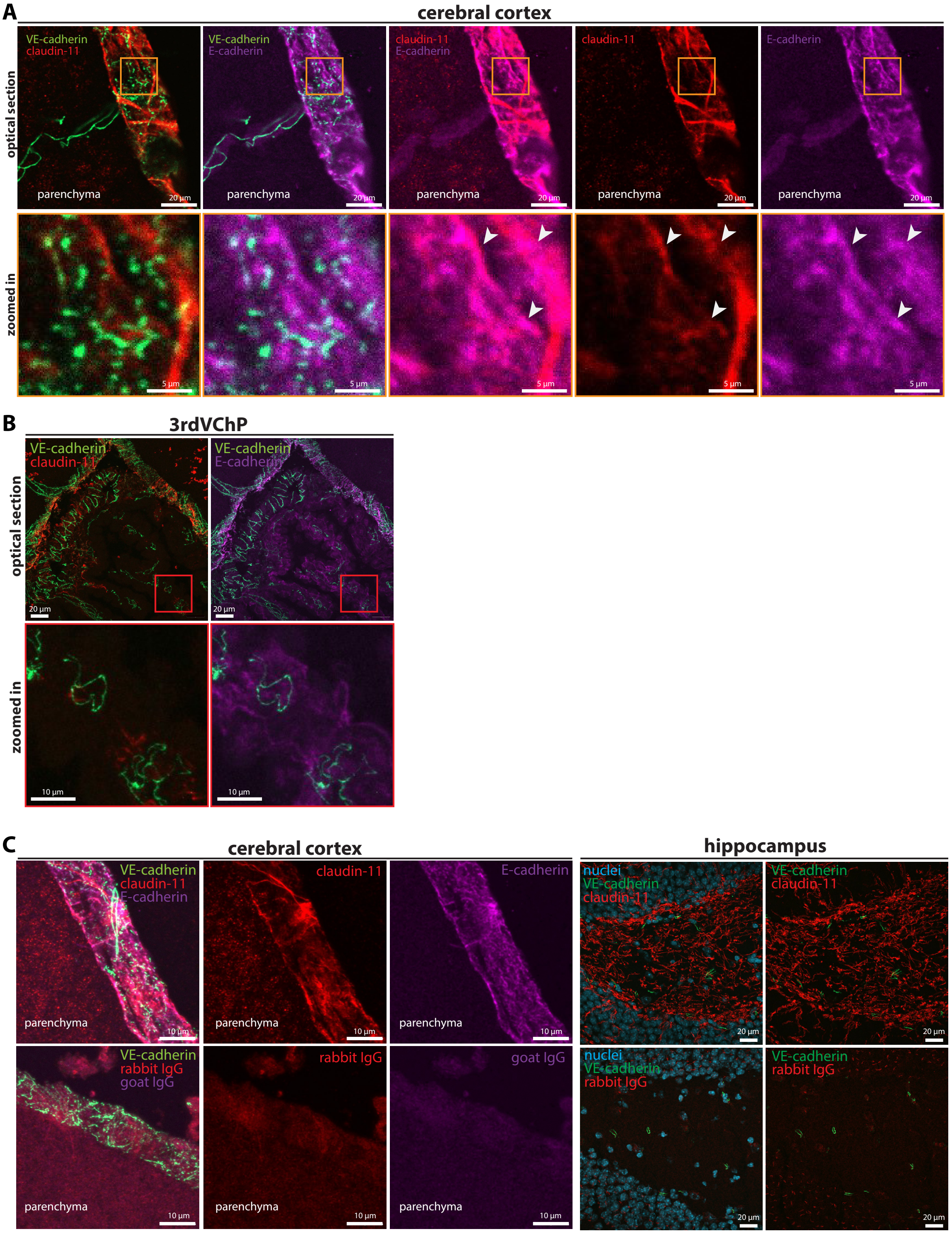
A
cerebral cortex
optical section
zoomed in
VE-cadherin
claudin-11
E-cadherin
parenchyma
20 µm
5 µm
B
3rdVChP
optical section
zoomed in
VE-cadherin
claudin-11
E-cadherin
20 µm
10 µm
C
cerebral cortex
hippocampus
VE-cadherin
claudin-11
E-cadherin
nuclei
rabbit IgG
goat IgG
parenchyma
10 µm
20 µm

Supplementary Figure 4. VE-cadherin-GFP and claudin-11 do not localize to the same arachnoid mater cell junctions

Immunofluorescence staining and confocal imaging of 20 µm thick sections of the brain from healthy VE-cadherin-GFP knock-in mouse immunostained for claudin-11 (red) and E-cadherin (magenta). **(A)** Representative images of the cerebral cortex shown as 0.16 µm optical sections, cropped and zoomed in on regions of interest. Claudin-11 staining partly colocalized with E-cadherin staining (white arrowheads). There is no visible overlap between VE-cadherin-GFP and claudin-11. Data is representative of three independent experiments. **(B)** Representative images of the third ventricle choroid plexus (3rdVChP) shown as 0.16 µm optical sections and cropped regions of interest. Surprisingly, Claudin-11 immunostaining did not detect choroid plexus epithelial cell tight junctions. Data is representative of three independent experiments. **(C)** Immunofluorescence staining for claudin-11 and E-cadherin and their respective isotype controls at the level of cerebral cortex and hippocampus. Data is representative of three independent experiments.

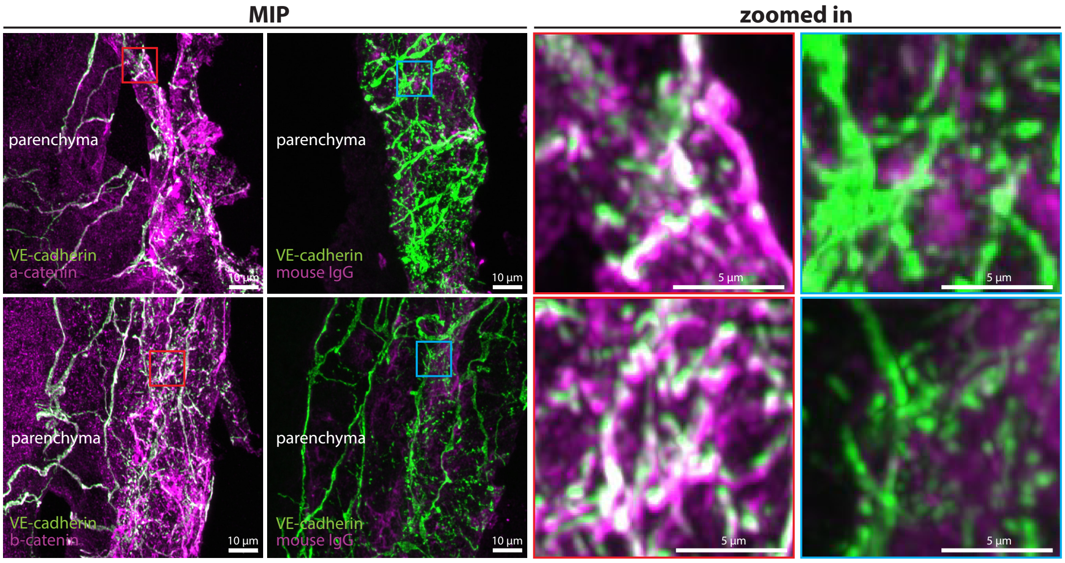

Supplementary Figure 5. VE-cadherin forms adherens junctions in leptomeningeal cells
Immunofluorescence staining of 20 µm thick sections of the brain from the healthy VE-cadherin-GFP (green) knock-in mouse immunostained for either α-catenin or β-catenin (magenta) and their respective isotype controls. Images are shown as maximal intensity projections (MIP) (left) and zoomed in regions of interest (right). Images acquired by a laser scanning confocal microscope equipped with the airyscan detector. Data is representative of three independent experiments.

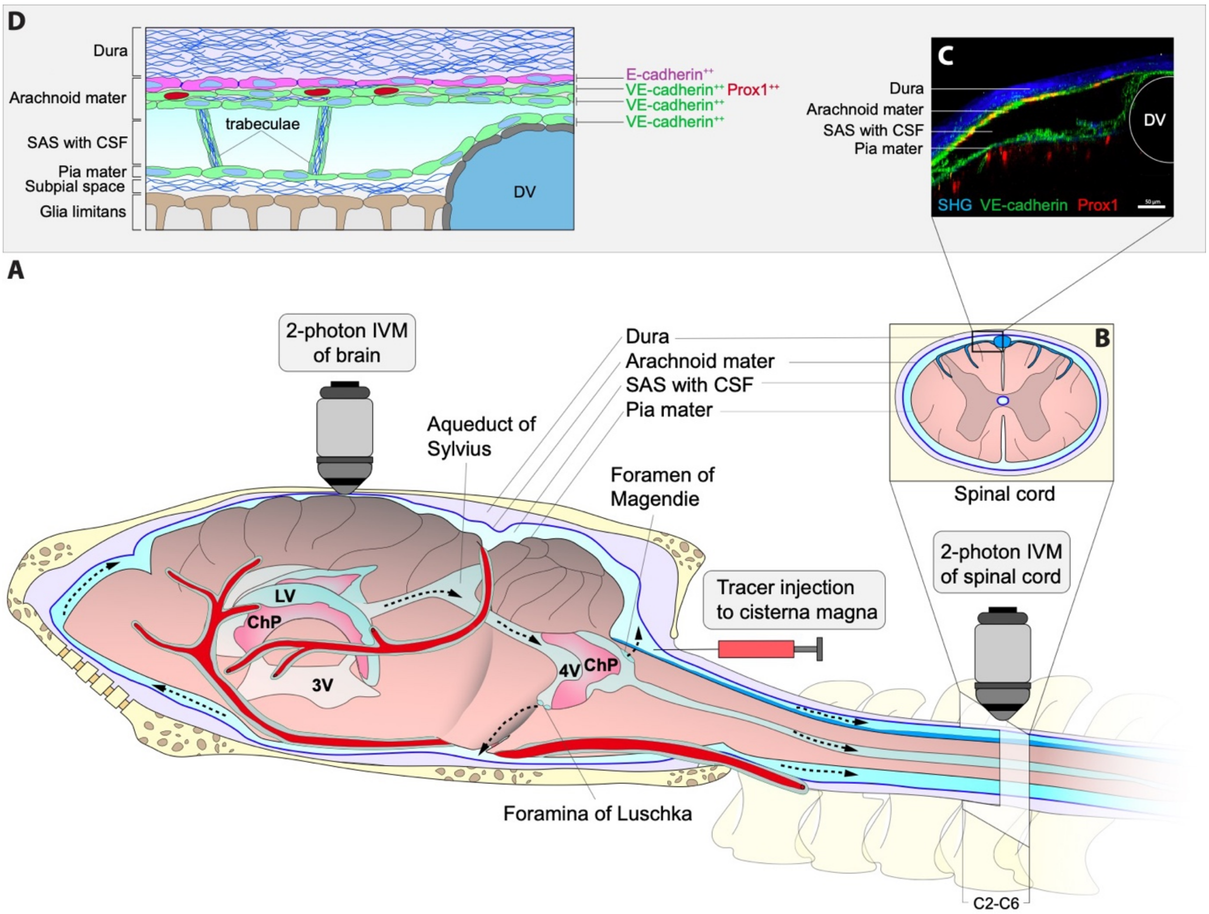

Supplementary Figure 6. Schematic representation of the CNS imaging approaches and spinal cord meninges
Schematic overview of the 2P-IVM in vivo imaging sites of the mouse brain and spinal cord performed in this study. Fluorescent tracers were infused into the cisterna magna to visualize cerebrospinal fluid (CSF) filled spaces (A). Routes of CSF bulk flow (dashed lines) are indicated to show the expected distribution of fluorescent tracers in CSF filled spaces (A). Different meningeal layers are shown on the coronal section of the mouse spinal cord (B). Image of the cervical spinal cord region adjacent to the dorsal vein (DV) of VE-cadherin-GFP; Prox1-tdTomato reporter mice acquired by in vivo 2-photon microscopy (C) and its schematic representation (D). SHG (blue) of the collagen fibers appears at the level of dura. The arachnoid mater is represented by distinct cellular layers: the upper, E-cadherin+ (magenta) arachnoid barrier cellular layer(s) interconnected by the tight junctions, and the lower, continuous VE-cadherin-GFP± (green) layers, partially coinciding with a Prox1+ (red) discontinuous cellular layer adjacent to the arachnoid barrier cells. The subarachnoid space (SAS) is bordered by VE-cadherin-GFP cells of arachnoid mater and pia mater, and occasionally crossed by trabeculae. Prox1-tdTomato cells localized below the pia mater are probably oligodendrocyte precursors (C). LV, lateral ventricle; ChP, choroid plexus; 3V, third ventricle; 4V, fourth ventricle; IVM, in vivo microscopy; C2-C6, cervical vertebrae 2-6.

Supplementary Table 1. List of Antibodies used in this study

Immunofluorescence Staining Antibodies			
**Primary Antibodies**	**Company**	**CAT Nr.**	**Working Conc.**
Rabbit anti-human/mouse E-cadherin	Cell Signaling	24E10.	10ug/ml
Rat anti-mouse /human /porcine ER-TR7	BMA Biomedical	T-2109	10ug/ml
Rat anti-mouse PECAM-1	In house	Clone MEC13.3	10ug/ml
Goat anti-human /mouse /rat /canine ALCAM	R&D Systems	AF1172	10ug/ml
Rabbit anti-mouse VE-cadherin	Provided by Prof. Dietmar Vestweber	VE-42	10ug/ml
Mouse anti-mouse /rat /dog /chicken α-catenin	BD	610194	10ug/ml
Mouse anti-mouse /human /rat /dog β-catenin	BD	610154	10ug/ml
Goat anti-mouse /human E-cadherin	R&D Systems	AF748	10ug/ml
Rabbit anti-mouse /human Claudin-11	Novus	NBP1-82470	10ug/ml
Mouse IgG1 k isotype	BioLegend	401402	10ug/ml
Rabbit IgG isotype	R&D Systems	AB-105-C	10ug/ml
Goat IgG isotype	R&D Systems	AB-108-C	10ug/ml
**Secondary Antibodies**	**Company**	**CAT Nr.**	**Working Conc.**
Donkey anti-rabbit IgG (H + L)-Cy5	Jackson ImmunoResearch	711-175-152	5ug/ml
Donkey anti-rat (H + L)-Cy3	Jackson ImmunoResearch	712-165-150	5ug/ml
Donkey anti-goat (H + L)-Cy3	Jackson ImmunoResearch	705-165-147	5ug/ml
Goat anti-mouse (H + L)-Alexa Fluor 647	Invitrogen	A32728	5ug/ml
Donkey anti-goat (H + L)-Alexa Fluor 647	Jackson ImmunoResearch	705-605-003	7.5ug/ml
