# Supplementary material for: VE-cadherin in arachnoid and pia mater cells serves as a suitable landmark for in vivo imaging of CNS immune surveillance and inflammation
Source: Nat Commun. 2023 Sep 20;14:5837. doi: 10.1038/s41467-023-41580-4 (PMC10511632; doi:10.1038/s41467-023-41580-4)
Supplement: Supplementary file 3 — Description of additional supplementary files [file 41467_2023_41580_MOESM3_ESM.pdf]

Description of additional supplementary files

Supplementary Movie 1. 2P-IVM imaging of the spinal cord of a healthy VE-cadherin GFP knockin mouse reveals morphological differences in VE-cadherin GFP expressing cells in the arachnoid and pia mater 3D reconstruction of the meningeal layers of the spinal cord. 2P-IVM imaging of the spinal cord of a healthy VE-cadherin-GFP knock-in reporter mouse after intravenous injection of dextran vascular tracer to visualize the blood vessels (red). The dura mater is visible in blue due to the SHG of the collagen type 1 fibers in the dura. VEcadherin-GFP is visible in green. VEcadherin AJs on the endothelial cells are visible in the blood vessel walls (red). Additional VE-cadherin GFP signal is visible under the dura mater characterized by VEcadherin GFP+ signal with junctional properties and no distinct cellular morphology. A second VE-cadherin GFP+ layer characterized by large cells with VEcadherin GFP+ AJs is seen at the level of the pia mater. The large SAS with trabeculae between the arachnoid barrier and pia mater is visible. This video is the source video of which images shown in Figure 1 B, D- G were taken. Images are representative of a total of 3 mice.

Supplementary Movie 2. Widening of a putative “subdural space” Time-lapse video of 2P-IVM imaging of the brain of a VE-cadherin GFP knock-in reporter mouse via a thinned skull preparation is shown. Time is shown in hours, minutes, and seconds. The bone and dura mater are visible in blue due to the SHG. Dural vessels, arachnoid mater and pia mater are visible in green due to their VE-cadherin-GFP expression. This video is the source video of which images shown in Supplementary Figure 1B were taken.

Supplementary Movie 3. Leptomeningeal VE-cadherinGFP+ adherens junctions display different patterns when compared to those of vascular endothelial cells A) Optical sectioning of a 300µm thick Zstack from 2P-IVM of a VE-cadherin-GFP knock-in mouse after skull thinning preparation and systemic injection of 10kDa dextran-AF647 (red). SHG depicts the bone and dura mater. Movie shows the optical sections starting from the top layer at the level of the bone to the bottom layer within the parenchyma. B) Optical sectioning of a 50µ thick transverse section from a decalcified head from a VEcadherin-GFP knock-in mouse previously perfused with Lectin-DyLight 594 (red). Movie shows the optical sections starting from the top layer at the expected level of the arachnoid mater to the bottom layer at the expected level of the pia mater and brain parenchyma.

Supplementary Movie 4. Prox1-TdTomato+ cells colocalize within VE-cadherin-GFP+ cells of the spinal cord arachnoid mater 3D reconstruction of the meningeal layers of the cervical spinal cord. 2P-IVM imaging of the cervical spinal cord of a healthy VEcadherin-GFP; Prox1-tdTomato double reporter mouse. The dura mater is visualized by SHG of the collagen type 1 fibers (blue). VE-cadherin-GFP is visible in green. Prox1-tdTomato is visible in red.

Supplementary Movie 5. The pia mater is a barrier for large molecular weight tracers Before the spinal cord window preparation, a tracer-filled cannula was implanted into the cisterna magna. During 2P-IVM, the mice were injected with 2.5µl of TRITC BSA at a rate of 1µl/min using a syringe pump 5mins after the start of 2PIVM. X–y–t time-lapse sequence of a 400 µm × 400 µm scan field at a depth of 160- 220 µm and 81-111 z-stacks with 2 µm spacing were acquired for 45mins after tracer injection. Time is shown in hours, minutes, and seconds. The dura mater is visible in blue due to the SHG of the collagen type 1

fibers in the dura. Arachnoid mater and pia mater are visible in green due to their VE-cadherin expression. Injected tracer is seen in red. At 5mins the SAS increases in size corresponding to the start of the CMI. At 45 mins, the TRITC BSA (red) is confined in the SAS. Scale bars = 50 µm. This video is the source video of which images shown in Figure 7A were taken.

Supplementary Movie 6. Cisterna magna injected 3kDa dextran rapidly drains into the periphery in neuroinflammation Time-lapse video of 2P-IVM imaging of the spinal cord of VE-cadherin GFP knock-in reporter mouse suffering from EAE (day 14 p.i., clinical score +). Before the spinal cord window preparation, a tracer-filled cannula was implanted into the cisterna magna. During 2P-IVM, the mice were injected with 2.5µl of 3kDa TRITC dextran at a rate of 1µl/min using a syringe pump. X–y–t time-lapse sequence of a 400 µm × 400 µm scan field at a depth of 160-220 µm and 81-111 z-stacks with 2 µm spacing were acquired for 45mins. Time is shown in hours, minutes, and seconds. The dura mater is visible in blue due to the SHG of the collagen type 1 fibers in the dura. Arachnoid mater and pia mater are visible in green due to their VE-cadherin expression. Injected tracer is seen in red. At 10 minutes, the 3kDa TRITC dextran (red) is seen in the dorsal vein and blood vessels in the sub arachnoid space (green) and 45 minutes the tracer (red) is seen above the dura mater (blue). Scale bars = 50 µm. This video is the source video of which images shown in Figure 8A were taken.

Supplementary Movie 7. Onset of EAE is accompanied by a significant enlargement of the spinal cord SAS and subpial space 3D reconstruction of the meningeal layers of the spinal cord. 2P-IVM imaging of the spinal cord of VE-cadherin GFP knock-in reporter mouse suffering from EAE (day 14 p.i., clinical score +), after intravenous injection of dextran vascular tracer to visualize the blood vessels (red). The dura mater is visible in blue due to the SHG of the collagen type 1 fibers in the dura. VEcadherin-GFP is visible in green. VEcadherin AJs on the endothelial cells are visible in the blood vessel walls (red). Additional VE-cadherin GFP signal is visible in the arachnoid mater, characterized by VE-cadherin GFP+ signal with junctional properties and no distinct cellular morphology, and on the pia mater, characterized by large cells with VEcadherin GFP+ AJs. The large SAS with trabeculae between the arachnoid barrier and pia mater is visible. Moreover, a subpial space is visible between the dorsal vein and the pia mater. This video is the source video of which images shown in Figure 9E were taken. Images are representative for a total of 3 mice imaged in a total of 3 experiments.

Supplementary Movie 8. Resolution of the inflammation at the peak of EAE 3D reconstruction of the meningeal layers of the spinal cord. 2P-IVM imaging of the spinal cord of VE-cadherin GFP knock-in reporter mouse suffering from EAE (day 25 p.i., clinical score +), after intravenous injection of dextran vascular tracer to visualize the blood vessels (red). The dura mater is visible in blue due to the SHG of the collagen type 1 fibers in the dura. VEcadherin-GFP is visible in green. VEcadherin AJs on the endothelial cells are visible in the blood vessel walls (red). Additional VE-cadherin GFP signal is visible in the arachnoid mater, characterized by VE-cadherin GFP+ signal with junctional properties and no distinct cellular morphology, and on the pia mater, characterized by large cells with VEcadherin GFP+ AJs. The large SAS with trabeculae between the arachnoid barrier and pia mater is visible. No subpial space is visible between the dorsal vein and the pia mater. This video is the source video of which images shown in Figure 9E were taken. Images are representative for a total of 3 mice imaged in a total of 3 experiments.

Supplementary Movie 9: The VE-cadherin GFP knock-in mouse allows for the assignment of CD8+ T-cells to the SAS 2P-IVM imaging of the spinal cord of a VEcadherin GFP knock-in mouse on day 7 after

induction of CD8+ T cell mediated neuroinflammation. OT-I CD8+ T cells visible in red. The pia mater is characterized by large cells with VE-cadherin-GFP+ AJs visible in green. Blue circles highlight the OT-I CD8+ T cells below the pia mater, and white circles highlight the OT-I CD8+ T cells that are above the pia mater. Scale bars =50 µm. This video is the source video of which images shown in Figure 10A were taken. Images are representative for a total of 7 mice imaged in a total of 7 experiments.

Supplementary Movie 10. Trafficking of CD8+ T cells across the leptomeningeal layers 2P-IVM imaging of the spinal cord of a VEcadherin GFP knock-in mouse on day 7 after induction of CD8+ T cell mediated neuroinflammation. OT-I CD8+ T cells visible in red. The pia mater is characterized by large cells with VEcadherin-GFP+ AJs visible in green. Cyan circles highlight the OT-I CD8+ T cells that are either above or below the pia mater or either potentially performing paracellular or transcellular diapedesis. Scale bars =50 µm. This video is the source video of which images shown in Figure 10 D-E were taken. Images are representative for a total of 7 VE-cadherin GFP mice and 7 ODC-OVA-xVE-cadherin GFP knock-in mice.
