# Supplementary material for: VE-cadherin in arachnoid and pia mater cells serves as a suitable landmark for in vivo imaging of CNS immune surveillance and inflammation
Source: Nat Commun. 2023 Sep 20;14:5837. doi: 10.1038/s41467-023-41580-4 (PMC10511632; doi:10.1038/s41467-023-41580-4)
Supplement: Supplementary file 14 — Reporting Summary [file 41467_2023_41580_MOESM14_ESM.pdf]

nature portfolio

Corresponding author(s): Britta Engelhardt

Last updated by author(s): Jul 24, 2023

Reporting Summary

Nature Portfolio wishes to improve the reproducibility of the work that we publish. This form provides structure for consistency and transparency in reporting. For further information on Nature Portfolio policies, see our Editorial Policies and the Editorial Policy Checklist.

Statistics

For all statistical analyses, confirm that the following items are present in the figure legend, table legend, main text, or Methods section.

n/a	Confirmed	
☐	☒	The exact sample size (*n*) for each experimental group/condition, given as a discrete number and unit of measurement
☐	☒	A statement on whether measurements were taken from distinct samples or whether the same sample was measured repeatedly
☐	☒	The statistical test(s) used AND whether they are one- or two-sided<br>*Only common tests should be described solely by name; describe more complex techniques in the Methods section.*
☒	☐	A description of all covariates tested
☐	☒	A description of any assumptions or corrections, such as tests of normality and adjustment for multiple comparisons
☐	☒	A full description of the statistical parameters including central tendency (e.g. means) or other basic estimates (e.g. regression coefficient) AND variation (e.g. standard deviation) or associated estimates of uncertainty (e.g. confidence intervals)
☐	☒	For null hypothesis testing, the test statistic (e.g. *F*, *t*, *r*) with confidence intervals, effect sizes, degrees of freedom and *P* value noted<br>*Give P values as exact values whenever suitable.*
☒	☐	For Bayesian analysis, information on the choice of priors and Markov chain Monte Carlo settings
☒	☐	For hierarchical and complex designs, identification of the appropriate level for tests and full reporting of outcomes
☒	☐	Estimates of effect sizes (e.g. Cohen's *d*, Pearson's *r*), indicating how they were calculated

Our web collection on statistics for biologists contains articles on many of the points above.

Software and code

Policy information about availability of computer code

Data collection	Confocal image acquisition was performed with the Zeiss LSM800 microscope, some images with a Zeiss LSM800 microscope equipped with an Airyscan, controlled by the Zen 2.6.76 software.<br>Two-photon image acquisition was performed with a TrimScope two-photon laser scanning microscopy (2PM) system (Mylteni; Germany) equipped with an Olympus BX50WI fluorescence microscope and a water immersion objective (20x, NA 0.95; Olympus). The 2PM system is controlled by ImSpector Pro64 v5.1.333 software (La Vision Biotech; Miltenyi). For two-photon excitation, a Ti:sapphire Spectra Physics InSight X3 laser was used. Distorsion correction of the images during two-photon microscopy imaging was performed with Vivo Follow 2.0 (Vladymyrov, M., Haghayegh Jahromi, N., Kaba, E., Engelhardt, B., Ariga, A. Vivo Follow 2: Distorsion-Free Multiphoton Intravital imaging. Frontiers in Physics. (2020)). Epifluorescence imaging was performed with the AxioZoom fluorescence microscope (Carl Zeiss, Oberkochen, Germany) equipped with a Plan Neo Fluar Z 1.0x/0.25 (7x to 112x Zoom) objective, controlled by the Zen 2.6. software. Transmission electron microscopy ultra-thin sections (60 nm) were cut on a UC7 ultramicrotome (Leica) and collected in nickel meshgrids coated with a Formvar film (Ted Pella). Sections were analyzed in a TecnaiTM G2 Spirit BioTwin at 80 kV.
Data analysis	Image analysis was performed using Image J/Fiji (version 2.3) and Imaris x64 9.8. Intravital microscopy cell tracks were obtained using the semiautomated spots function, manually corrected from Imaris x64 9.8.<br>Statistical analysis and data representation was performed with GraphPad Prism v9.2.<br>Figures were made with Adobe Illustrator 25.4.1.<br>Movies were processed with Adobe After Effects 18.4.1.

For manuscripts utilizing custom algorithms or software that are central to the research but not yet described in published literature, software must be made available to editors and reviewers. We strongly encourage code deposition in a community repository (e.g. GitHub). See the Nature Portfolio guidelines for submitting code & software for further information.

Data

Policy information about availability of data

All manuscripts must include a data availability statement. This statement should provide the following information, where applicable:

- Accession codes, unique identifiers, or web links for publicly available datasets
- A description of any restrictions on data availability
- For clinical datasets or third party data, please ensure that the statement adheres to our policy

All quantitative data is made available as a Source Data file. Raw imaging data will be made available in the public repository ImmuneMap or upon request.

Research involving human participants, their data, or biological material

Policy information about studies with human participants or human data. See also policy information about sex, gender (identity/presentation), and sexual orientation and race, ethnicity and racism.

Reporting on sex and gender	n/a
Reporting on race, ethnicity, or other socially relevant groupings	n/a
Population characteristics	n/a
Recruitment	n/a
Ethics oversight	n/a

Note that full information on the approval of the study protocol must also be provided in the manuscript.

Field-specific reporting

Please select the one below that is the best fit for your research. If you are not sure, read the appropriate sections before making your selection.

☒ Life sciences ☐ Behavioural & social sciences ☐ Ecological, evolutionary & environmental sciences

For a reference copy of the document with all sections, see nature.com/documents/nr-reporting-summary-flat.pdf

Life sciences study design

All studies must disclose on these points even when the disclosure is negative.

Sample size	A minimum of three mice per experiment were analyzed in each experiment unless otherwise stated in the figure legend. For the in vivo cell tracking we analyzed 30 cells per mice and condition. No power calculation was performed, however, the sample size was sufficiently high to show statistical differences in the observed effects.<br>For all the in vivo imaging experiments, a minimum of three independent experiments with three different mice per condition were performed. Specific sample sizes for each experiment are detailed in the figure legends.<br>The number of mice imaged per condition and cells counted per FOV are within the ranges of comparable in vivo imaging studies in the field. Specific details are included in the figure legends.
Data exclusions	We did not exclude any data from consideration
Replication	All the results reported in this study were successfully reproduced with the minimum of three biological replicates.
Randomization	For all experiments, randomization was applied among mice with the specific genotype and disease score required in each experiment.
Blinding	Complete blinding of the experimenters was not possible since specific mouse genotypes were required in each experiment. Taking this into account, data analysis was regularly double-checked by at least one additional experimenter.

Reporting for specific materials, systems and methods

We require information from authors about some types of materials, experimental systems and methods used in many studies. Here, indicate whether each material, system or method listed is relevant to your study. If you are not sure if a list item applies to your research, read the appropriate section before selecting a response.

Materials & experimental systems

n/a	Involved in the study
☐	☒ Antibodies
☒	☐ Eukaryotic cell lines
☒	☐ Palaeontology and archaeology
☐	☒ Animals and other organisms
☒	☐ Clinical data
☒	☐ Dual use research of concern
☒	☐ Plants

Methods

n/a	Involved in the study
☒	☐ ChIP-seq
☒	☐ Flow cytometry
☒	☐ MRI-based neuroimaging

Antibodies

Antibodies used	Detailed information about the antibodies used in this study are provided in the methods section and in supplementary table 1.
Validation	All antibodies used are commercially available and have been validated by their manufacturers. All relevant data regarding their validation can be found in their webpages using the catalog numbers provided in supplementary table 1, along with all the relevant information about the antibodies used.

Animals and other research organisms

Policy information about studies involving animals; ARRIVE guidelines recommended for reporting animal research, and Sex and Gender in Research

Laboratory animals	Female mice from the C57BL/6J strain or VE-cadherin-GFP C57BL/6J or ODC-OVA C57BL/6 mice between 6-12 weeks were used in this study. Species, strains, sex and age of all laboratory animals used in this study are also adequately reported in the subsection "Mice" of the "Methods" section in the manuscript. All mice were housed in individually ventilated cages under specific pathogen free conditions in a temperature-controlled room (22°C) with a 13:11 hours light:night cycle. They had ad libidum access to water and chow.
Wild animals	No wild animal was used in this study.
Reporting on sex	Only female mice were used in this study due to higher reproducibility of the neuroinflammation models in female mice as well as to avoid possible immune reactions in the ODC-OVA, VE-cadherin-GFP mice after the T cell transfer from different donors from different sex.
Field-collected samples	No field-collected sample was used in this study.
Ethics oversight	Animal procedures were approved by the Veterinary Office of the Canton Bern (permit no BE31/17, BE77/18 and BE98/20) and are in line with institutional and standard protocols for the care and use of laboratory animals in Switzerland.

Note that full information on the approval of the study protocol must also be provided in the manuscript.
